# Food and Water Safety Risks From Salmonella enterica and Escherichia coli Contamination in Bukavu City, Democratic Republic of the Congo

**DOI:** 10.7759/cureus.79951

**Published:** 2025-03-03

**Authors:** Alain M Okito, John M Wagacha, Catherine W Lukhoba, Alex A Lina, Wolfgang R Mukabana

**Affiliations:** 1 Department of Biology, Université Officielle de Bukavu, Bukavu, COD; 2 Department of Biology, University of Nairobi, Naiorbi, KEN; 3 Department of Biology, University of Nairobi, Nairobi, KEN

**Keywords:** bacterial infectious disease, bukavu restaurants, escherichia coli, food safety practices, one health approach, public and environmental health, salmonella enterica

## Abstract

Introduction

Bukavu city is facing sanitation challenges, with many reported cases of epidemics such as cholera, salmonellosis, and other infectious diseases. Food and water sold under poor sanitation conditions are exposed to potential contamination by pathogenic bacteria. Consumers are therefore at risk of contracting bacterial infections, which could increase the burden of infectious diseases. Solid waste and wastewater management is unsatisfactory in Bukavu city. Few scientific investigations have been conducted to assess the influence of environmental conditions on food and water contamination. For these reasons, this study aimed to investigate the quality of food sold in restaurants and on the streets, as well as the types of water and milk produced and consumed, since food safety and environmental sanitation remain major public health challenges in Bukavu city.

Objective

The broad objective of this study was to assess the safety risks associated with food and water contamination by *Salmonella enterica* and *Escherichia coli* in Bukavu city, DR Congo.

Methods

A field observation was conducted to identify restaurant locations, and a probabilistic model was applied to determine the number of restaurants to be surveyed. A simple random method was then applied to survey 206 food handlers in restaurants across all three municipalities of Bukavu city. A total of 415 food samples from restaurants, 34 milk samples from dairy farms, and 104 water samples from taps, tanks, and wells were collected. *Salmonella enterica* and *Escherichia coli* were isolated from these samples, characterized, and identified through cultural methods and biochemical tests.

Results

The presence of *Salmonella enterica *was observed in 38.2% of pork, sausage, and ugali samples (n = 34 for each food), 35.5% of bread, 32.4% of beef and potatoes (n = 34 for each food), and 24.4% of beans (n = 41) tested positive for *S. enterica*. While *Escherichia coli* was not isolated from beans, beef, potato, sausage, or vegetable samples, the bacterium was most prevalent in fish (11.8%, n = 34). More than half (57.1%, n = 28) of the water samples from wells were contaminated by *S. enterica*, and 35.7% (n = 28) by *E. coli*. The high rate of well contamination could be explained by the location of wells near public waste disposal sites, sewage systems, and pathways frequently used by humans and animals. The presence of *S. enterica *and *E. coli *was not significantly different in food (p = 0.15) and water (p = 0.58), suggesting that the environmental conditions of Bukavu city are favorable for the growth of both bacterial species in food and water. However, food contamination by *S. enterica *and *E. coli *was significantly (p < 0.05) dependent on food handling practices such as "frequency of cooking food" and "heating food before serving customers" in both formal and informal restaurants (street-vended food).

Conclusion

Hygiene practices in Bukavu restaurants were not sufficient to guarantee safe food for human consumption, particularly during the serving stages. There is a need for public health and environmental decision-makers to develop or update guidelines in line with national and international standards to enhance public health countermeasures in and around wells, restaurants, and street-vended food and to improve water treatment and infrastructure in Bukavu city.

## Introduction

*Salmonella enterica* and *Escherichia coli* are among the *Enterobacteriaceae* that cause infectious diseases in humans and animals. These species live in water, food, soil, grass, and air and are implicated in foodborne and waterborne diseases [1,2]. Food and water are therefore the main sources from which pathogenic bacteria spread from the environment to infect humans and animals. Worldwide, *Salmonella* infection remains a public health threat, with 11.9 million typhoid fever cases and approximately 129,000 deaths per year in low- and middle-income countries. This issue is exacerbated by poverty due to the heavy economic burden of treatment [3].

The Democratic Republic of Congo, a low-income country, faces the challenge of foodborne and waterborne diseases, along with multifaceted conflicts and other health crises [4,5]. These challenges contribute to population displacement, high mortality rates, and increasing cases of emerging and re-emerging diseases [6,7]. Indeed, contaminated food and water pose a threat to human and animal health by causing various bacterial and parasitic infections [8,9]. However, the prevalence of bacteria in environmental samples such as food and water is underestimated. Previous studies have primarily focused on the prevalence of *Salmonella *and *Escherichia *infections in human samples (blood, urine, and stool) collected from hospitals [10-12]. The environmental aspects of infections associated with these pathogens are often neglected or under-investigated.

Residents of Bukavu city struggle to access clean drinking water despite the presence of Lake Kivu and several rivers [13]. In recent years, the Inspectorate of Hygiene and Health Conditions has reported several cases of diarrhea, enteric fever, cholera, and typhoid fever in the city [9]. A study in Bukavu city revealed a high incidence of *E. coli *(83%) and other pathogenic bacterial species (77%) in samples from street-vended foods [14]. Given that these foods are more accessible to lower-income populations, they constitute an important but often overlooked factor in the transmission of pathogenic bacteria. For example, Ngaruka et al. reported an association between the type of food consumed and the prevalence of *Salmonella *infection in patients [15].

In the Bukavu hinterlands, farmers produce milk and other dairy products such as cheese and yogurt, but the microbial quality of these products remains a challenge [16]. In addition to inadequate hygiene practices in food handling, poor sanitation remains a major public health challenge in Bukavu city. The city lacks sufficient sewage systems to ensure the safe collection and disposal of wastewater [6,13]. In some neighborhoods, water shortages can last for a week, while in others, there is no piped water supply. Water is therefore obtained from natural or artificial wells or from Lake Kivu [6]. Additionally, Bukavu city experiences various challenges, including landslides, water pollution, recurring cholera outbreaks, the presence of displaced populations, and impassable roads [9,13].

These challenges, combined with unreliable water supply and insufficient hygiene practices in restaurants, increase consumers' susceptibility to foodborne and waterborne diseases. This could silently contribute to a rise in infectious diseases in Bukavu city. However, there are limited studies on the prevalence of foodborne and waterborne diseases and the occurrence of *S. enterica* and *E. coli* in Bukavu. The public health challenge is further exacerbated by insufficient food and water safety controls by state services.

In alignment with the One Health concept, which promotes a holistic approach to addressing health issues as defined by Fao et al. [17], this study aimed to assess the hygienic conditions of restaurants and street-vended ready-to-eat foods, determine the prevalence of *S. enterica *and *E. coli *in food, milk, and water samples, and evaluate the distribution of *S. enterica *and *E. coli *in food in relation to hygiene practices applied by food handlers in restaurants. By achieving these objectives, this study has contributed to raising awareness among Bukavu residents. The findings could encourage food handlers to systematically monitor hygiene practices, particularly during food service. Additionally, the study provides recommendations for public health authorities to strengthen routine food and water quality monitoring. Finally, this study serves as scientific evidence to urge local and national decision-makers to improve sanitation in Bukavu city.

## Materials and methods

Study area 

**Figure 1:**
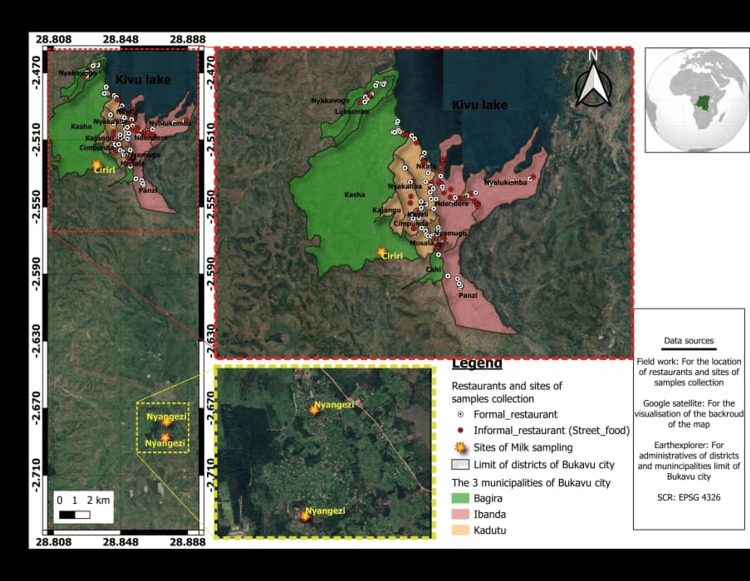
Sampling sites across three municipalities of Bukavu city where food and water samples were collected and two localities (Ciriri and Nyangezi) for milk samples. This figure is the authors' own creation using the global positioning system of each location where samples were collected. This map was created using QGIS. The waypoints were collected using KoboCollect.

The food, water, and milk samples were collected in Bukavu city, located at 2º29’-2º33’ South and 28º48’-28º52’ East, at an altitude ranging from 1,463 to 2,200 m in South Kivu Province, Democratic Republic of Congo. The city spans 60 km² and has approximately 1.5 million inhabitants residing in three municipalities: Bagira, Ibanda, and Kadutu (Figure 1). The Bukavu region is characterized by a humid tropical climate, with nine months of rainfall (September-May) and three dry months (June-August), receiving an average annual rainfall of 1,414 mm [9,13].

Field observations and determination of sample size for the survey

The selection of restaurants for investigation was based on the geological morphology of Bukavu city, which is dominated by mountains, with most economic activities concentrated along the main roads. Given the lack of official statistics on the number of restaurants, a field observation was conducted to locate restaurants across the three municipalities of Bukavu city. A total of 119, 321, and 302 formal restaurants and street-vended food establishments were identified in Bagira, Ibanda, and Kadutu, respectively. A survey questionnaire was used to collect information from food handlers. Before administering the questionnaire to respondents, a pre-survey was conducted from November 10 to 17, 2022, in selected restaurants located in the Kharale district, an area primarily frequented by students from three universities: Université Officielle de Bukavu, Université Catholique de Bukavu, and Institut Supérieur des Techniques Médicales de Bukavu. Following the pre-survey, the questionnaire was refined to improve clarity and relevance.

To determine the number of food handlers to be surveyed, a probabilistic model was applied using the equation:

$n=\frac{Ɛ^{2}pq}{d^{2}}$

where *n* represents the sample size, *p* is the proportion of hygiene practices applied in restaurants, *q* is 1 - *p*, *Ɛ* is 1.65, and *d* is the precision at 10%. Given that the proportion of restaurants and their hygiene practices in Bukavu were unknown, *p* and *q* were assigned equal probabilities (*p* = 50% and *q* = 50%) [18]. By substituting these values into the equation, the minimum number of food handlers required for the survey was determined to be 67.

Field survey 

To select the restaurants to be included in the study, each municipality was considered a subgroup of all restaurants in Bukavu city, forming three subgroups based on the field observation: 119, 321, and 302 restaurants in Bagira, Ibanda, and Kadutu, respectively. Next, a simple random method was applied to identify which restaurants to include in the study. Using a survey questionnaire, food handlers were interviewed in two stages, from December 10 to 20, 2022, and from June 5 to 23, 2023. The structured questionnaire was implemented in the Kobo Collect application as a tool to collect data related to the socio-demographic profile of food handlers, characterization of restaurants and hygiene practices, sanitation conditions in and around restaurants, and the origin of water used for cooking and drinking in restaurants (Tables 4-6). One food handler was interviewed in each restaurant, and the same questionnaire was used for all participants.

During the pre-survey, most food handlers refused to participate in the study, requesting payment before responding to the questions. Others refused for personal reasons or due to being too busy serving customers. To minimize refusals, the survey involved visiting restaurants and engaging the food handler by explaining the purpose of the study before requesting their participation. If a food handler refused to respond, the investigator moved to the next identified restaurant to survey as many restaurants as possible. Using this approach, 234 food handlers were interviewed, and information related to the study objectives was collected. During data processing, all questionnaires where respondents did not answer at least three questions were excluded from the study, leaving responses from 206 food handlers for statistical analysis (Tables 1, 2). The simple random method was also applied to identify taps, wells, and tanks for water sampling. Milk samples were collected from two localities surrounding Bukavu city.

**Table 1:** Categorization of restaurants in each municipality (data from our survey). A formal restaurant is the one registered and paying taxes to the townhall, local or national government while an informal restaurant is neither registered nor paying taxes.

Municipality	Type of restaurants		Total
	Formal restaurant	Informal restaurant	
Bagira	32	9	41
Ibanda	41	53	94
Kadutu	46	25	71
Total	119	87	206

**Table 2:** Categorization of restaurants according to the price of meals across Bukavu municipality. The price of meal was categorized according to the DR Congo classification as low-income country by the World Bank [4].

Municipality	Price of meals		Total
	Low-cost restaurant (≤ 1 $)	Expensive restaurant (>1 $)	
Bagira	40	1	41
Ibanda	85	9	94
Kadutu	70	1	71
Total	195	11	206

Collection and transportation of samples for bacteriological analyses

Five hundred and fifty-three (553) samples, consisting of 415 food samples, 104 water samples, and 34 milk samples, were collected and examined (Table 3) from December 2022 to January 2024. Water and milk samples were collected in the morning from 6:00 to 9:00 hours, while food samples were collected from 10:00 AM to 14:00 hours. Approximately 100 g of each food sample was purchased from restaurants; 500 mL of water was sampled from each tap, tank, or well; and 500 mL of each milk sample was collected from dairy farms. Samples were placed in sterile Erlenmeyer flasks and transported in a cooler box within four hours post-sampling to the Microbiology and Biotechnology Laboratory of the “Université Officielle de Bukavu” for bacterial isolation, biochemical tests, and species identification.

**Table 3:** Sample collection sites and number of food, milk, and water samples analyzed. Kabare, Ciriri, and Nyangezi are among suburbs and territories surrounding Bukavu city.

Municipality	Food	Water			Milk	Total
		Tap	Tank	Well		
Bagira	65	12	4	4	0	85
Ibanda	105	16	0	4	0	125
Kadutu	245	16	16	20	0	297
Kabare	0	0	12	0	0	12
Ciriri	0	0	0	0	13	13
Nyangezi 1	0	0	0	0	11	11
Nyangezi 2	0	0	0	0	10	10
Total	415	44	32	28	34	553

Isolation and identification of *Salmonella enterica* and *Escherichia coli* from samples

*S. enterica *and *E. coli *were isolated according to Adzitey et al. [19] with modifications. For *S. enterica*, 25 g of food or 25 mL of water/milk sub-samples were aseptically weighed, pre-enriched in 225 mL of peptone water (PW) (TM Media, India), homogenized, and incubated at 37°C for 24 hours. Subsequently, 1 mL of positive PW culture was transferred into 10 mL of Tryptone Water Broth (TWB) (HiMedia, India) and incubated at 37°C for 24 hours. Next, 1 mL of positive TWB culture was inoculated onto Hektoen Enteric Agar (HEA) (TM Media, India) and incubated at 37°C for 24 hours. Finally, 1-3 presumptive *Salmonella* colonies from HEA were streaked onto Salmonella-Shigella (SS) agar (TM Media, India) and incubated at 37°C for 24 hours. If no colony grew on SS agar, plates were re-incubated under the same conditions to confirm the true absence of *Salmonella*. For *E. coli*, 1 mL of positive PW culture was inoculated onto Eosin Methylene Blue (EMB) agar and incubated at 37°C for 24 hours. Suspected *Escherichia *colonies from EMB were then streaked onto MacConkey agar (MCA) and incubated at 44°C for 24 hours. From SS agar or MCA, 2-3 colonies were streaked onto nutrient agar (Oxoid, England) for purification and incubated at 37°C for 24 hours. Finally, purified *Salmonella *or *Escherichia *isolates underwent morphological characterization through macroscopic observation of colonies grown on selective agar and microscopic observation using Gram staining. For biochemical characterization, isolates were tested for citrate, catalase, indole, urease, glucose and lactose fermentation, H₂S production, and motility according to Rahman et al. [20].

Statistical analysis 

Data were analyzed using RStudio 4.4.0 [21]. Descriptive statistics were applied to compute the prevalence of bacteria based on the occurrence of S. enterica or E. coli in positive cultures and to summarize data related to the socio-demographic characteristics of food handlers. Additionally, the number of "yes/presence" (indicating the application of hygiene practices in restaurants) and "no/absence" (indicating non-application of hygiene practices) was recorded for each question in the questionnaire. The distribution of bacteria in each type of food was assessed in relation to the responses (yes or no) to hygiene practices provided by food handlers. Next, a contingency table was created, and the chi-square test was applied to determine the dependence or independence of the occurrence of *S. enterica *and *E. coli *in food samples relative to the application or non-application of hygiene practices by food handlers. The binomial test at a 95% confidence interval (CI) was applied to assess the probability and reliability of bacterial prevalence in food, water, and milk, as well as the proportion of other qualitative variables assessed during the survey. Mean differences in each test were considered significant at p-value ≤ 0.05.

Ethical considerations

Prior to conducting this study, the proposal was presented during a special postgraduate seminar and approved by the Postgraduate Committee of the University of Nairobi in Kenya and the *Université Officielle de Bukavu* in DR Congo. During the field activity, participation in the survey was voluntary. Each food handler was informed about the questionnaire and the purpose of the study, provided with verbal assurance of the strict confidentiality of the information collected, and asked for verbal consent before participating in the study.

## Results

Demographics of food handlers

Out of the 206 food handlers, 90.8% were under 50 years old, 54.9% were male, and 64.6% had a literacy level up to secondary school. The distribution of food handlers by age, marital status, and literacy level was significantly different (p < 0.05), but no significant difference was observed based on gender (p = 0.29) (Table 4).

**Table 4:** Demographics of food handlers (n = 206) from the surveyed restaurants in Bukavu city, DR Congo, in 2023. The chi-square test was used to compare the distribution of food handlers against demographic variables (age, gender, marital status, literacy level). Mean differences were considered significant at p-value ≤ 0.05 with confidence interval (CI) at 95%.

Variables	Frequency (n = 206)	% (95%CI)	p-value	Chi-square value
Age				
15–50 years old	187	90.8 (85.9-94.3)	0.00	96.9
>50 years old	19	9.2 (5.6-14.0)		
Gender				
Female	93	45.1 (38.2-52.2)	0.29	1.1
Male	113	54.9 (47.8-61.8)		
Marital status				
Single	86	41.7 (34.9-48.8)	0.00	138.5
Married	109	52.9 (45.9-59.9)		
Divorced	4	2 (0.5-4.9)		
Widowed	7	3.4 (1.4-6.9)		
Literacy level				
Illiterate	19	9.2 (5.6-14.0)	0.00	185.1
Primary	32	15.5 (10.9-21.2)		
Secondary	133	64.6 (57.6-71.1)		
Undergraduate	21	10.2 (6.4-15.2)		
No response	1	0.5 (0.0-2.8)		

Hygiene practices and types of foods sold in Bukavu restaurants

After analyzing responses to each questionnaire, the survey revealed that most of the 206 food handlers followed hygiene practices that did not ensure sufficient food safety conditions. In particular, many restaurants lacked hand sanitizers and washrooms, were surrounded by garbage, and sold food exposed to dust. Most restaurants also lacked refrigerators for food storage. Despite this, they served food cooked the day before, and some did not reheat food before serving customers. Waitresses did not wear a hat or appropriate uniform during food service. The majority of restaurants used water supplied by the state company but did not boil it before serving customers (Table 5). Additionally, 12 types of food were commonly sold and consumed in Bukavu restaurants, including ugali, vegetables, beans, bread, potatoes, rice, fish, beef, pork, sausage, samosas, and peanuts (Table 6).

**Table 5:** Hygiene practices by food handlers in Bukavu restaurants, DR Congo, in 2023. Chi-square was applied to assess the distribution of food handlers against hygiene practices applied in restaurants. Mean differences were considered significant at p-value ≤0.05, with a confidence interval (CI) at 95%.

Variables	Frequency (n = 206)	% (95% CI)	p-value	Chi-square value
Source of food raw materials				
Directly from farm and fishery	2	1.0 (0.1-3.5)	0.00	272.3
Public market	179	86.9 (81.5-91.2)		
Grocery store or supermarket	9	4.3 (2.0-8.1)		
Other sources	16	7.8 (4.5-12.3)		
Mode of transportation of food raw materials				
Walking with food on head	77	37.4 (30.8-44.4)	0.01	8.9
Vehicle (taxi, bus, car)	83	40.3 (33.5-47.3)		
Motorcycle	46	22.3 (16.8-28.6)		
Type of restaurant customers				
Children	3	1.5 (0.3-4.2)	0.00	107.6
Adults	62	30.1 (23.9-36.9)		
Children and adults	141	68.4 (61.6-74.7)		
Availability of hand sanitizer				
Yes	84	40.8 (34.0-47.8)	0.00	76.0
No	115	55.8 (48.8-62.7)		
Present sometimes	7	3.4 (1.4-6.9)		
Availability of hand washing device				
Yes	128	62.1 (55.1-68.8)	0.00	7.6
No	78	37.9 (31.2-44.9)		
Presence of washroom in restaurant				
Yes	117	56.8 (49.7-63.7)	0.13	2.27
No	89	43.2 (36.3-50.3)		
Garbage around restaurants				
Yes	142	68.9 (62.1-75.2)	0.00	19.2
No	64	31.1 (24.8-37.9)		
Food exposed to dust				
Yes	139	67.5 (60.6-73.8)	0.00	85.3
No	52	25.2 (19.5-31.7)		
Sometimes	15	7.3 (0.4-11.7)		
Food on continuous heat				
Yes	103	50.0 (43.0-57.0)	0.00	29.5
No	72	35.0 (28.5-41.9)		
Sometimes	31	15.0 (10.5-20.7)		
Food preservation state before serving				
Food stored open	68	33.0 (26.6-40.0)	0.00	15.3
Food stored closed	138	67.0 (60.1-73.4)		
Frequency of cooking food				
Daily	179	86.9 (81.5-91.2)	0.00	359.5
Once per week	3	1.5 (0.3-4.2)		
Twice per week	7	3.4 (1.4-6.9)		
Thrice per week	12	5.8 (3.0-10)		
Others (depending on sales)	5	2.4 (0.8-5.6)		
Wearing uniforms during food service				
Yes	10	4.9 (2.4-8.7)	0.00	121.1
No	196	95.1 (91.3-97.6)		
Refrigerate food at the restaurant				
Yes	35	17.0 (12.1-22.8)	0.00	61.5
No	171	83.0 (77.2-87.9)		
Offer takeaway food service				
Yes	133	64.6 (57.6-71.1)	0.00	11.1
No	73	35.4 (28.9-42.4)		
Packaging type used for take-way food				
Nylon bag	1	0.5(0.0-2.7)	0.00	91.5
Plastic bag	104	50.5(43.5-57.5)		
Other packaging (carton, kraft paper)	36	17.4(12.5-23.4)		
No response	65	31.6(25.3-38.4)		
Overnight food served				
Yes	139	67.5 (60.6-73.8)	0.00	16.3
No	67	32.5 (26.2-39.4)		
Heating food before serving customers				
Yes	102	49.5 (42.5-56.5)	0.00	18.5
No	45	21.9 (16.4-28.1)		
No response	59	28.6 (22.6-35.3)		
Water used and consumed in the restaurant				
REGIDESO water	168	81.5 (75.6-86.6)	0.00	234.1
Bottle packaged water	23	11.2 (7.2-16.3)		
Water from well	1	0.5 (0.0-2.7)		
Water not served to customers	14	6.8 (3.8-11.1)		
Water boiled before serving customers				
Yes	9	4.4 (2.0-8.1)	0.00	124.0
No	197	95.6 (91.9-98)		

**Table 6:** Occurrence of S. enterica and E. coli in food, water, and milk samples collected in Bukavu city, DR Congo, in 2023. ^a^Samosa is a popular pastry that is typically triangular in shape filled with spiced vegetables, potatoes, or meat, and is deep-fried until crispy. ^b^Ugali is a staple food in Eastern and Central Africa made from maize or cassava flour mixed with boiling water to form a thick porridge and is typically served with meat or vegetable stews. The chi-square test was applied to assess whether the occurrence of *S. enterica* and *E. coli *was different in each type of food, water, and milk. Mean differences were considered significant at p-value ≤0.05. The confidence interval (CI) of 95% was applied to estimate the probability of *S. enterica* and *E. coli *occurrence in food, water, and milk.

Sample type	*S. enterica*, n (%)	(95% CI)	*E. coli*, n (%)	(95%CI)	p-value	Chi-square value	
							Food (n = 415)							
Beans (n = 41)	10 (24.4)	(12-4 0)	0 (0.0)	(0-8.6)	0.15	15.57	
Beef (n = 34)	11 (32.4)	(17.4-50.5)	0 (0.0)	(0-10.3)			
Fish (n = 34)	9 (26.5)	(12.9-44.4)	4 (11.8)	(3-27.5)			
Bread (n = 34)	12 (35.3)	(19.7-53.5)	2 (5.9)	(1-19.7)			
Peanuts (n = 34)	8 (23.5)	(10.7-41.2)	2 (5.9)	(1-19.7)			
Pork (n = 34)	13 (38.2)	(22.2-56.4)	1 (2.9)	(0-15.3)			
Potato (n = 34)	11 (32.4)	(17.4-50.5)	0 (0.0)	(0-10.3)			
Rice (n = 34)	7 (20.6)	(8.7-37.9)	1 (2.9)	(0-15.3)			
Samosa^a^(n = 34)	6 (17.6)	(6.8-34.5)	2 (5.9)	(1-19.7)			
Sausage (n = 34)	13 (38.2)	(22.2-56.4)	0 (0.0)	(0-10.3)			
Ugali^b ^(n = 34)	13 (38.2)	(22.2-56.4)	2 (5.9)	(1-19.7)			
Vegetables (n = 34)	9 (26.5)	(12.9-44.4)	0 (0.0)	(0-10.3)			
Total (n = 415)	122 (29.4)	(25.1-34.0)	14 (3.4)	(2-5.6)			
Water (n = 104)							
Tap (n = 44)	3 (6.8)	(1.4-18.7)	2 (4.5)	(0.6-15.5)	0.58	1.07	
Tank (n = 32)	5 (15.6)	(5.3-32.8)	1 (3.1)	(0.1-16.2)			
Well (n = 28)	16 (57.1)	(37.2-75.5)	10 (35.7)	(18.6-55.9)			
Total (n = 104)	24 (23.1)	(15.4-32.4)	13 (12.5)	(6.8-20.4)			
Milk (n = 34)							
Farm 1 (n = 13) from Ciriri	1 (7.7)	(0.2-36.0)	0 (0.0)	(0-25.0)	0.07	3.46	
Farm 2 (n = 11) from Nyangezi	2 (18.2)	(2.0-52.0)	0 (0.0)	(0-28.0)			
Farm 3 (n = 10) from Nyangezi	3 (30.0)	(7.0-65.0)	0 (0.0)	(0-31.0)			
Total (n = 34)	6 (17.6)	(7.0-35.0)	0 (0.0)	(0-10.0)			

Prevalence of *Salmonella enterica* and *Escherichia coli* in food, water, and milk 

**Figure 2:**
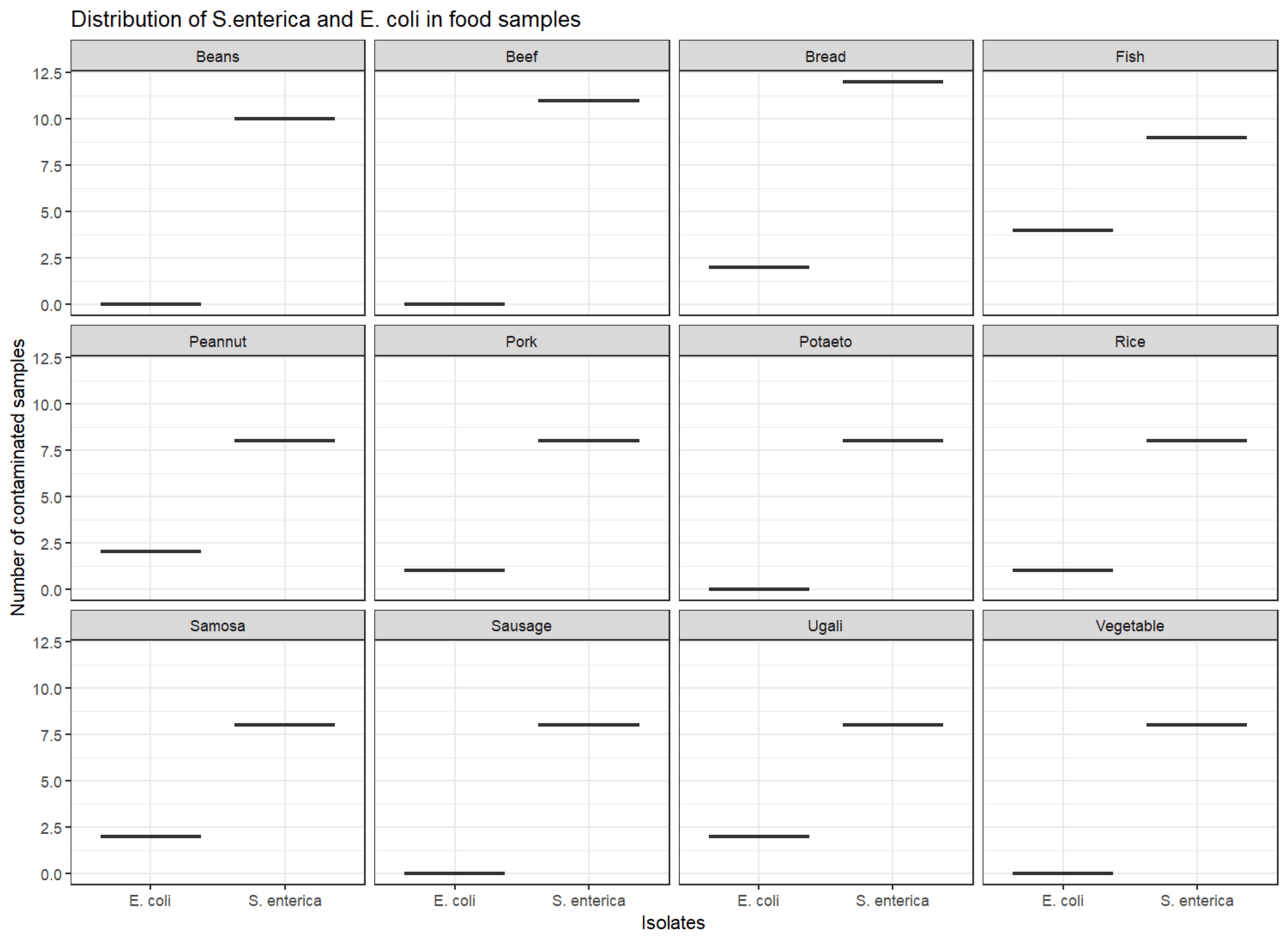
Distribution of S. enterica and E. coli isolated from 12 types of food most consumed in restaurants of Bukavu city. This figure summarizes the number of positive samples for *S. enterica *and *E. coli* in each food sample.

**Figure 3:**
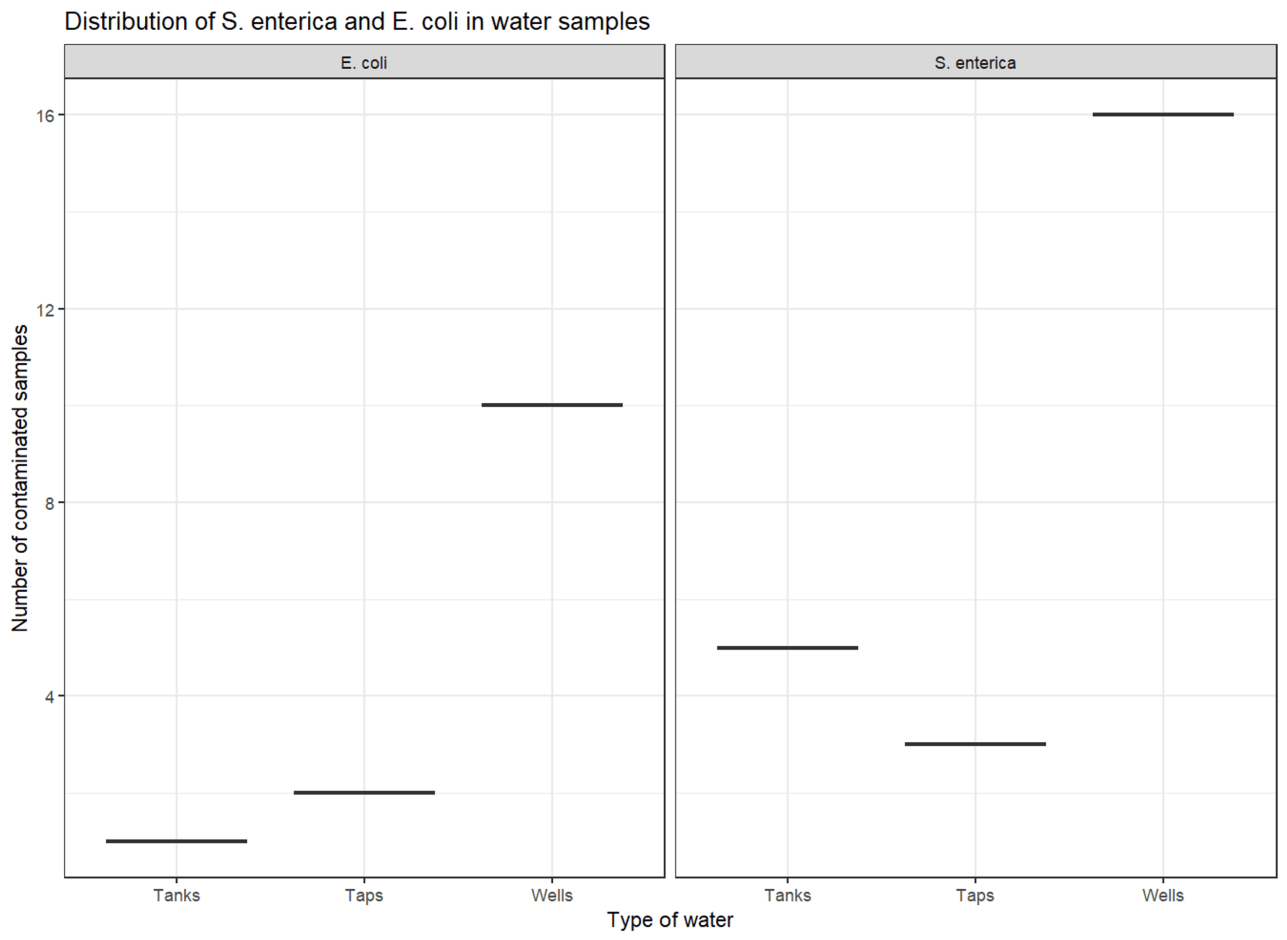
Distribution of S. enterica and E. coli isolated from three types of water analyzed in Bukavu city. This figure summarizes the number of positive samples for *S. enterica* and *E. coli *in each water sample. Water from taps and tanks is treated and distributed by the state company. Wells are natural or artificial excavations in the ground with flowing water.

Table 6 presents the distribution of *S. enterica *and *E. coli *strains in food, water, and milk samples. The prevalence of *S. enterica *was high in pork (38.2%, n = 34), sausage (38.2%, n = 34), ugali (38.2%, n = 34), bread (35.3%, n = 34), beef (32.4%, n = 34), potatoes (32.4%, n = 34), and beans (24.4%, n = 41). While *E. coli *was not isolated from beans, beef, potatoes, sausage, or vegetable samples, the bacterium was most prevalent in fish (11.8%, n = 34) (Figure 2). Water from wells (n = 28) tested positive for *S. enterica* (57.1%) and *E. coli *(35.7%) (Figure 3). Milk samples from Nyangezi (farms 2 and 3) were positive for *S. enterica *(23.8%, n = 21), while none tested positive for *E. coli*. Contamination by *S. enterica *and *E. coli *did not significantly differ between food (p = 0.15) and water samples (p = 0.58), suggesting that the occurrence of both bacteria was independent of the type of food and water analyzed. The presence of *Salmonella* in milk samples was also independent (p > 0.05) of the location of the dairy farms (Ciriri or Nyangezi).

Distribution of *Salmonella enterica *and *Escherichia coli *in food samples in relation to hygiene practices in Bukavu restaurants

There was a significant (p ≤ 0.05) relationship between the distribution of *S. enterica *and *E. coli *strains in food and the hygiene practices of "heating food before serving customers," "frequency of cooking food," and "type of water used in the restaurant." Statistical analysis showed that other hygiene practices, such as "availability of hand sanitizer," "availability of a handwashing device," "availability of washrooms," "presence of garbage around restaurants," "exposure of food to dust," "state of food preservation before serving," and "refrigeration of food in restaurants," also contributed to bacterial contamination in food but with a non-significant probability (p > 0.05). The distribution of *S. enterica *and *E. coli *in food was not associated with the "location of restaurants" (Bagira, Ibanda, Kadutu), "type of restaurant" (formal or informal), or the "price of meals" (cheap or expensive) (p > 0.05) (Table 7).

**Table 7:** Association between hygiene practices and the occurrence of S. enterica and E. coli in food samples collected in Bukavu city, DR Congo, in 2023. The chi-square test was applied to assess if the distribution of *S. enterica *and *E. coli *in food and water samples was in relation to hygiene practices applied in restaurants. Mean differences were considered significant at p-value ≤0.05.

Independent variables	Dependent variables		p-value	Chi-square value
	*S. enterica*, n (%) (N = 122)	*E. coli*, n (%) (N = 14)		
Municipality				
Bagira	22 (18.0)	1(7.1)	0.15	3.68
Ibanda	37 (30.3)	2 (14.3)		
Kadutu	63 (51.6)	11(78.6)		
Type of restaurant				
Formal	41 (33.6)	5(35.7)	1.00	0.00
Informal	81 (66.4)	9(64.3)		
Price of meals				
Cheap	89 (73.0)	11(78.6)	0.89	0.01
Expensive	33 (27.0)	3 (21.4)		
Availability of hand sanitizer				
Yes	20 (16.4)	2 (14.3)	0.97	0.04
No	68 (55.7)	8 (57.1)		
Sometimes	34 (27.9)	4 (28.6)		
Availability of hand washing device				
Yes	46 (37.7)	3 (21.4)	0.36	0.82
No	76 (62.3)	11(78.6)		
Presence of washroom in restaurant				
Yes	69 (56.6)	9(64.3)	0.78	0.07
No	53 (43.4)	5(35.7)		
Garbage around restaurants				
Yes	84 (68.9)	11(78.6)	0.65	0.19
No	38 (31.1)	3(21.4)		
Food exposed to dust				
Yes	82 (67.2)	9(64.3)	0.81	0.41
No	11 (9.0)	2(14.3)		
Sometimes	29 (23.8)	3(21.4)		
Food on continuous heat				
Yes	58 (47.5)	7(50.0)	0.96	0.08
No	43 (35.2)	5(35.7)		
Sometimes	21 (17.2)	2(14.3)		
Food preservation state before serving				
Food stored opened	83 (68.0)	11(78.6)	0.61	0.25
Food stored closed	39 (32.0)	3(21.4)		
Frequency of cooking food				
Daily	56 (45.9)	12(85.7)	0.03	10.66
Once per week	9 (7.4)	0(0.0)		
Twice per week	6 (4.9)	0(0.0)		
Thrice a week	15 (12.3)	1(7.1)		
Others (depending on food sales)	36 (29.5)	1(7.1)		
Wearing uniforms during food service				
Yes	24 (19.7)	1(7.1)	0.43	0.61
No	98 (80.3)	13(92.9)		
Refrigerate food at the restaurant				
Yes	21 (17.2)	1(7.1)	0.55	0.34
No	101 (82.8)	13(92.9)		
Overnight food served				
Yes	82 (67.2)	9(64.3)	1.00	0.00
No	40 (32.8)	5(35.7)		
Heating food before serving customers				
Yes	27 (22.1)	7(50.0)	0.05	5.98
No	60 (49.2)	3(21.4)		
No response	35 (28.7)	4(28.6)		
Type of water used in the restaurant				
State company water	78 (63.9)	11(78.6)	0.03	8.55
Bottle package water	28 (23.0)	0(0.0)		
Water of well	3 (2.4)	2(14.3)		
Water not served to customers	13 (10.7)	1(7.1)		
Water boiled and served to clients				
Yes	117 (95.9)	13 (92.8)	0.65	0.20
No	5 (4.1)	1 (7.2)		

## Discussion

Prevalence of *Salmonella enterica* and *Escherichia coli* in food, water, and milk samples

The findings from the current study revealed a notable prevalence of *S. enterica* and *E. coli* across different food, water, and milk samples collected in Bukavu city, which could be attributed to various environmental and hygiene-related factors. The study noted a prevalence of 29.4% for *S. enterica* and 3.4% for *E. coli *among 415 food samples. Among water samples (n = 104), 23.1% tested positive for *S. enterica* and 12.5% for *E. coli. *Out of 34 milk samples, 17.6% tested positive for *S. enterica*. These findings highlight the public health risks associated with these pathogenic contaminants in Bukavu city.

The contamination levels observed in this study are consistent with findings from other regions where poor sanitation and waste management systems have been identified as contributing factors to bacterial contamination. A study conducted in Bukavu city confirmed that all street-vended food samples (n = 80) tested positive for *S. enterica* (100%) and *E. coli* (100%), which could increase the risk of infectious diseases and pose a health burden for low-income populations [14]. Other studies have reported that Bukavu city continues to struggle with waste and sanitation issues [9,13]. Consequently, food and water sold under such environmental conditions remain highly susceptible to contamination by pathogens, including *S. enterica *and *E. coli*.

Focusing on the bacterial prevalence in each of the 12 analyzed food types, *S. enterica* ranged from 17.6% (n = 34) in samosas to 38.2% (n = 34) in pork, sausage, and ugali. The prevalence of *E. coli* ranged from 2.9% (n = 34) in pork and rice to 11.8% (n = 34) in fish. These results align with those reported by Ombeni et al. [14], who found that all street-vended foods (sausage, boiled meat, fish) were contaminated with *S. enterica* and *E. coli*.

Moreover, a study conducted in Kinshasa, DR Congo, reported that the prevalence of S. enterica was 11.1% (n = 72) in fish, 14.4% (n = 84) in meat sampled from slaughterhouses, 18.3% (n = 120) in meat from public markets, and 27.5% (n = 40) in beef carcasses from public abattoirs [22]. In Motta town, Ethiopia, Yasigat et al. [23] reported a *Salmonella *prevalence of 2.5% (n = 255) among food handlers. In Assiut, Egypt, El-Mohsen et al. [24] reported a prevalence of 11.4% (n = 220) for *S. enterica* in chicken.

In related studies, *S. enterica* was detected in vegetable salads (8.5%, n = 246) and in raw mixed salads (2.6%, n = 306) [25], in 47.5% (n = 225) of meat samples [19], and in poultry (13.9%), pig (13.1%), and cattle fecal samples (5.3%) [26]. The high prevalence of S. enterica and *E. coli* in food could be attributed to several factors, including improper food handling. Additionally, the health status of waiters and waitresses may influence food contamination, particularly among those who do not undergo regular medical check-ups [23].

Similar to the findings of the current study regarding food and water, results from other studies indicate a high prevalence of *S. enterica *in human and animal samples in the DRC. The prevalence of *S. enterica* was reported in human stool samples (4.4%, n = 98) and blood samples (30.4%, n = 299) [10,11], as well as in rats (8.1%, n = 566) [27]. This suggests that food and water remain a neglected niche for *S. enterica* and *E. coli*, from which humans and animals could become infected. The study underscored the role of hygiene practices in food safety, particularly in restaurant environments and during food service stages. Therefore, there is a need to establish strong control measures and countermeasures to mitigate the spread of pathogenic bacteria and reduce cases of infectious diseases in low-income settings such as the DR Congo.

Water samples from wells (n = 28) were more contaminated with *S. enterica* (57.1%) and *E. coli *(35.7%) than water from taps and tanks. A previous study reported a high prevalence of parasites (57%-77%) in rivers crossing Bukavu city [9], while Mbuyi-Kalonji et al. [28] found that the prevalence of parasites in the environment was correlated with the presence of non-typhoidal *Salmonella *(NTS) in co-infection with Schistosoma mansoni. In Butembo city, DR Congo, 40% of the water distributed by the state company did not meet World Health Organization standards for human consumption due to bacterial contamination, including *E. coli *and *S. enterica *[8]. However, the current study differs from the findings of Nieniea et al. [29], who did not isolate *E. coli *from well water samples collected in Kikwit city, DR Congo. Milk samples from Nyangezi (farm 3) were more contaminated (30%, n = 10) with *S. enterica *than those from Ciriri (farm 1, n = 13) and Nyangezi (farm 2, n = 11). These findings are consistent with those of Bacigale et al. [16], who reported a high prevalence of *S. enterica *(66.7%, n = 302), while *E. coli *was not detected in milk samples.

The current study suggests that water sources, particularly wells, pose a greater contamination risk for *S. enterica *and *E. coli*. Milk contamination may result from inadequate hygiene practices during collection and storage, compromising the bacterial quality of milk sold in Bukavu city. Poor water and milk quality pose a health risk to humans and animals. Addressing this issue requires strengthening existing water treatment plants with enhanced microbiological treatment protocols, improving sanitation around wells, and enforcing better hygiene practices during milk collection.

Distribution of *S. enterica* and *E. coli *strains in food samples in relation to hygiene practices 

This study assessed the distribution of *S. enterica *and *E. coli *strains in food samples in relation to hygiene practices applied in restaurants to determine the probable origin of bacterial contamination in ready-to-eat food sold in Bukavu restaurants. The results revealed that *S. enterica *and *E. coli *were present in food samples collected from restaurants, indicating inadequate hygiene practices that make food unsafe for consumers. Ombeni et al. [14] and Yasigat et al. [23] reported similar findings, showing that the occurrence of pathogenic bacteria in street-vended foods was strongly influenced by poor hygiene among food handlers. Additionally, food contamination was associated with poor personal hygiene, including untrimmed and unclean fingernails, *Salmonella* carriers among food handlers, the absence of proper clothing or footwear during service, and improper utensil cleaning practices.

The association between poor hygiene and the presence of these pathogens is evident. Combined with inadequate food handling and poor personal cleanliness among food handlers, the risk of food contamination in Bukavu restaurants remains difficult to control. This underscores the need for improved sanitation and hygiene protocols, particularly in public food establishments and markets where food is often exposed to contamination.

This study did not account for seasonal variations in the contamination of milk, food, and water samples by *Salmonella *and *Escherichia *species. However, Tack et al. [30] reported that non-typhoidal *Salmonella *(NTS) occurrence in the DR Congo was associated with rainfall. This suggests that environmental factors and climate change may contribute to the spread of NTS. Therefore, the findings highlight the necessity of controlling environmental dimensions of food safety to limit the spread of *Salmonella *and *Escherichia *species in Bukavu city. Moreover, it is essential to train farmers and food handlers on proper handling of milk, food, and water. In addition, the Public Health Authority must enhance surveillance and reinforce routine checks of food, water, and milk quality before public consumption.

Socio-demographic profiles of food handlers in Bukavu restaurants

Regarding the socio-demographic profiles of food handlers, the study found that the majority were under 50 years old, male, married, and had a secondary school education. These findings corroborate studies conducted in Bukavu city [14] and Motta town, Ethiopia [23], regarding the education level and age of food handlers and their limited training in food safety. In contrast, 84% (n = 80) of food sellers were women from villages surrounding Bukavu city [14], while in Motta town, 82.7% (n = 243) of food handlers were women [23]. Despite these differences, the distribution of food handlers in the current study based on gender was not statistically significant (54.9% versus 45.1%, p = 0.29). Regardless of these factors, hygiene practices remain below acceptable standards, emphasizing the need for food safety training and capacity-building programs.

Additionally, the study revealed that the distribution of *S. enterica* and *E. coli *was independent of restaurant type, food price, or location. This suggests that all food establishments are at risk of contamination, particularly when raw materials are sourced from public markets with potentially compromised hygiene standards. If hygiene practices are neglected during food preparation and handling, contaminants can survive these stages, posing serious health risks to consumers. Furthermore, the unregulated construction in Bukavu city and the lack of an adequate waste management system contribute to severe sanitation challenges, resulting in direct environmental contamination and indirect contamination of food and water.

Limitations of the study

The results of this study highlighted some realities of Bukavu city and its hinterlands. Foodborne and waterborne diseases being a countrywide challenge, other studies in Bukavu city and data from other regions or cities of DR Congo should be collected and analyzed before generalizing these findings. Another limitation of this study was the absence of molecular analysis of the isolated bacteria. Molecular analysis could have allowed the study of genomic sequences. This would open up further investigations on pathogenic bacteria isolated from food, water, and milk samples, to compare them with those isolated from human and animal samples in DR Congo. Also, the prevalence of pathogenic bacterial genes in food and water samples should have been determined. Furthermore, this study did not consider seasonal variations linked to the presence of bacteria in food and water samples. This could have revealed another factor in the prevalence of these pathogens. In addition, there is a need for future studies to develop a model for qualitative and quantitative microbiological risk assessment to improve food and water checking in Bukavu city, DR Congo.

## Conclusions

This study has revealed significant concerns regarding hygiene practices in Bukavu restaurants, which operate under insufficient hygiene measures that compromise food safety. The high prevalence of *S. enterica* and *E. coli *in food, milk, and water samples indicates a considerable risk of infectious diseases to consumers. This study underscores that food and water are often neglected environmental sources that silently harbor pathogenic bacteria, contributing to their spread throughout Bukavu city. This represents a significant public health challenge. Public Health Authorities in Bukavu must, therefore, prioritize enhanced surveillance and stricter monitoring of food and water quality. Developing clear guidelines and countermeasures aligned with national standards and adopting a comprehensive approach, such as One Health, is essential. Additionally, more focus should be placed on minimizing bacterial contamination during food handling and serving stages, particularly in street-vended and ready-to-eat foods. This study calls for improved sanitation practices, better waste management, and the implementation of stronger regulatory frameworks for food safety and water treatment. Educating food handlers and the public could significantly reduce the health burden of foodborne and waterborne diseases in Bukavu city.
